# The Story of the Hospitals

**Published:** 1887-07-23

**Authors:** 


### THE STORY OF THE HOSPITALS.

### GUY'S HOSPITAL.

*(Concluded from p. 260, vol. ii.)*

THERE are a number of small rooms in this building used by the electricians of the hospital, and here come daily large numbers of people suffering from paralysis, in its various forms, to be "dosed"—if the term may be used—from the electric battery. The appliances here are of the latest standard of excellence, the operator having at his hand a number of buttons and handles, by means of which double or single currents of various degrees of strength may be produced.

The Operating Theatre.

The operations with the electric current here carried on remind us that we have overlooked, in our description of the old building, the operating theatre on the second floor. It was the least pleasing sight of the whole inspection. There was something very grim in the little silent room, with its untenanted table in the middle, and its rows of seats for the students perched high above each other to the roof. Yet a moment's reflection showed how little these feelings of repulsion were justified, and induced rather a feeling of thankfulness that the discovery of anæsthetics has enabled many a painful operation to be performed without the patient being conscious of the touch of the knife, and that the extension of surgery thus rendered possible has saved many a precious life. *Experientia docet* is a maxim which holds good of the surgical more than of any other calling, and the gentlemen who witness these operations are graduating in a profession than which none is more distinctly a glory of the human race. Opposite the operating theatre is a room where the instruments used in the surgical wards are kept in order. Patriotic readers will be glad to know that the superiority which the French so long enjoyed in this department of manufacture has passed away, and that English makers are, as they should be, to the fore.

New Premises.

In the new building, before alluded to, are the out-patients' departments, the medical wards, the ophthalmic rooms, the anatomical museum in connection with the medical school, and, at the very top of the house, the nurses' dormitories. This building presents a marked contrast to the other section of the hospital which was built by Thomas Guy himself. The latter has an old-fashioned air; its staircases are narrow, and its stairs of wood, whilst its carved oak balustrades put one more in mind of an old country-house than of a refuge for the sick and suffering of modern Babylon. The new building, on the contrary, is modern from bottom to top. It is constructed of iron and stone, and, though hardly so picturesque, is admirably adapted for the purpose for which it was built. Unfortunately some of the medical wards are empty for lack of funds; and another, which has been set apart for paying patients, the charge to whom is £3 3*s.* per week besides the fees of the medical attendants, had only two occupants. That the management of Guy's Hospital is severely practical, was evidenced by the appearance of a ward which was under the decorator's hands. No delicate shades were to be seen on the walls, and no attempt at ornamentation was anywhere observable. A plain, clean-looking stone colour, picked out with dark red—this was all the design. "The walls can be easily washed, and will not want repainting for ten or twelve years," we were told in answer to a question.

"Sisters."

The sisters' room at the end of the ward had been embellished by some friendly hand with paintings of vines and ivy which clambered over the mantelpiece and sprawled in studied disorder round the window even up to the ceiling. *A propos* of the term "sisters," a word or two of explanation may be given here. The word suggests to most minds a religious, semi-conventual association, but the connection of the sisters with the hospital, or with the profession of nursing in general, is a purely commercial one. It is true that in the old ecclesiastical hospitals the nurses were really members of a sisterhood, and the term "sisters" having been used by Thomas Guy in his will, has been used in the hospital ever since its foundation. But in many similar institutions the appellation is quite a modern one, and in the provinces has not yet quite displaced the old designation of "nurse." In Guy's, as in most other hospitals in London, the "sisters" are taken from the ranks of the lady pupils who enter the hospital nursing classes, the fees from which, in this particular case, add some thousand pounds a year or so to the fund.

About the Out-Patients.

We have written at such length of the internal arrangements of the hospital, that but little space is left for a reference to the out-patients' department. Upon the knotty and much-disputed question whether the operations of the London hospitals in this direction have a beneficial effect upon the moral well-being of the London poor, we will not enter. Suffice it to say that at Guy's, at all events, the belief prevails that absolutely free medical advice and medicine given indiscriminately, is not the proper method to pursue; and a system has been adopted, the operation of which, up to the present, has not been attended with any evil results, nor productive of any serious complaint on the part of the people themselves. When a patient presents himself or herself for the first time, he sees the doctor and receives his medicine without any fee, but on the next and following weeks a charge of threepence is made for the medicine. If the patient declares, as some of them do, that he "hasn't threepence in the world," he is provided with a form, which he is directed to take to the nearest office of the Charity Organisation Society. That body sends one of its officers to inquire into the case, and it is surprising in how few instances it is found that the weekly payment of threepence is beyond the patient's means. A similar payment is enforced when urgent cases come to be treated in the old building—a department to which we referred in the previous article. The same system runs all through the hospital. Surgical or accident cases which require crutches, on leaving the institution, only receive those articles without charge when absolute destitution is proved to exist; otherwise a deposit of 2*s*. is made, to be returned when the crutches are returned.

About the In-Patients.

Acting on the same principle, in-patients who can afford it, but whose means will not permit them to enter the paying wards, are charged a guinea a week, and in many cases the sum is willingly paid by the employers of the sick or injured patients. Exception has been taken to the last-named charge on the ground that people who can pay so much ought to be treated at their own homes by regular practitioners, upon whom, it is alleged, the system presses somewhat harshly; but the reply of the hospital authorities is pretty decisive on this point. These cases, or the vast majority of them would, they allege, certainly come to the hospital in any event, and the hospital is perfectly justified in recouping itself of a portion of the cost of their maintenance. We have stated the arguments *pro* and *con.*, and must leave our readers to form their own judgment. It only remains to add that in the year 1885-6 the hospital received from in- and out-patients of all classes £2,376; and when it is considered that every year the expenditure exceeds the receipts by some thousands of pounds, this addition to the funds is more than welcome.

The Out-Patients Again.

But to return to the out-patients. It surprised us to learn that during the hopping season the number fell off very considerably. "Surely," we asked, "people who are so ill that they have to come to a hospital for advice and medicine can't do much good in the hop fields." "Well, you see," responded the woman of whom our inquiry was made, and who was evidently an *habituée* of the waiting-room, "such as we can't afford to lose a job, and if money's to be had, it's no use being ill." To which one of the doctors added that the fresh air of Kent would probably do more for them than all the physic in the dispensary. As it happened to be the very middle of the hopping season when we visited the hospital, and as, moreover, it was a women's day, there was not so much bustle and life in this department as is usually the case, and the cheerful-looking person who presides at the "bar," where tea, coffee, milk, buns, etc., are retailed at a most moderate price, had very little to do. Still we saw enough to convince the most sceptical of the vast work which is performed so unostentatiously here week after week and year after year. Another form which the activity of the out-patients' department takes is in the direction of attending poor women at their confinements. The lying-in charity in connection with the hospital confines its operations strictly within a mile radius of the hospital. Yet more than 2,500 women are annually relieved by this means—a striking testimony to the enormous mass of poverty which crowds itself outside the walls of this great hospital.

*(To be continued.)*

